# Teachers’ Beliefs About Children’s Anger and Skill in Recognizing Children’s Anger Expressions

**DOI:** 10.3389/fpsyg.2020.00474

**Published:** 2020-03-24

**Authors:** Courtney A. Hagan, Amy G. Halberstadt, Alison N. Cooke, Pamela W. Garner

**Affiliations:** ^1^Department of Psychology, North Carolina State University, Raleigh, NC, United States; ^2^School of Integrative Studies, George Mason University, Fairfax, VA, United States

**Keywords:** emotion, beliefs, emotion recognition, teacher-student relationship, emotion understanding

## Abstract

Everyday beliefs often organize and guide motivations, goals, and behaviors, and, as such, may also differentially motivate individuals to value and attend to emotion-related cues of others. In this way, the beliefs that individuals hold may affect the socioemotional skills that they develop. To test the role of emotion-related beliefs specific to anger, we examined an educational context in which beliefs could vary and have implications for individuals’ skill. Specifically, we studied 43 teachers’ beliefs about students’ anger in the school setting as well as their ability to recognize expressions of anger in children’s faces in a dynamic emotion recognition task. Results revealed that, even when controlling for teachers’ age and gender, teachers’ belief that children’s anger was useful and valuable in the school setting was associated with teachers’ accuracy at recognizing anger expressions in children’s faces. The belief that children’s anger was harmful and not conducive to learning, however, was not associated with teachers’ accuracy at recognizing children’s anger expressions. These findings suggest that certain everyday beliefs matter for predicting skill in recognizing specific emotion-related cues.

## Introduction

Emotion-related beliefs are thought to play an integral role in organizing individuals’ behaviors, motives, and goals (Lozada et al., 2016; De Castella et al., 2018; Oertwig et al., 2019). Beliefs may also play a crucial role in interpersonal relationships, as they likely guide individuals in understanding the emotions of others, and in deciding whether to approach or avoid the emotional content they express. Indeed, associations between emotion-related beliefs and subsequent behavior and skill have been demonstrated in a variety of studies, particularly in the field of parenting (Halberstadt et al., 2008; Dunsmore et al., 2009).

Despite research linking beliefs to behaviors in the parenting world, we know little about how beliefs function when adults are engaged in other roles or how beliefs might be specific to particular emotions. For example, we know very little about teachers’ emotion-related beliefs, despite their importance as educators and socializers of young children. The beliefs that teachers bring into the school setting every day may well be relevant to the ways in which teachers engage in their instructional practices and support the socioemotional tenor of the classroom. Teachers’ beliefs may also be specific to the classroom environment, which generally involves one adult and many children, and the multiple responsibilities of keeping the many children in that setting not only safe, but also educationally engaged.

In the current study, we were particularly interested in exploring teachers’ beliefs about anger as a specific emotion relevant within the classroom. First, teachers might have differing views about anger, as some individuals appraise anger as “unsafe” or harmful in interpersonal relationships, others perceive anger as serving no adaptive function, and others view anger as an opportunity to motivate positive action (Averill, 1982; Tavris, 1982; Miller and Sperry, 1987; Leonard et al., 2011). Second, teachers are encouraged to explain or offer direct instruction about appropriate ways of expressing and managing negative affect, often without their own clarity of what they believe about the emotion (Yelinek and Grady, 2019). Anger might also be an emotion about which teachers have some ambivalence, thinking that low-level anger in students might best be ignored and left unidentified in order to avoid having to issue consequences which might then escalate anger further. Thus, although teachers may widely share the goals of fostering children’s academic, social, and intrapsychic growth, they might have different types of emotion-related beliefs, which then differentially guide their attention to and skill in understanding emotion in the classroom.

Understanding others’ emotions is considered to be a foundational skill of emotional competence that allows for assessing and anticipating the behavior of others, and then adapting one’s own behavior to achieve one’s interpersonal goals (Mayer et al., 2001; Hall et al., 2009; Castro et al., 2016). Although children, or even adults, do not often show what they are feeling in highly prototypical ways (Reisenzein et al., 2013; Castro et al., 2018; Barrett et al., 2019), teachers need to accurately monitor students’ emotions during their many individual and classroom-level interactions throughout the day (Garner et al., 2019a). Further, teachers and other childcare professionals who understand students’ emotions seem to more effectively manage children’s emotions and classroom behaviors, and enjoy strong, positive relationships with students (Hargreaves, 2000; Demetriou and Wilson, 2010; Garner et al., 2019b; Valente et al., 2019). Thus, we tested whether teachers’ beliefs about anger might have implications for teachers’ understanding of students’ emotions, and specifically, teachers’ recognition of anger expressions as they develop in children’s faces.

### Teachers’ Beliefs About Emotions

Early work on teachers’ beliefs about emotions focused on teachers’ beliefs about whether and how early childcare teachers should instruct children about emotional expression (Hyson and Lee, 1996). Beliefs about *how* emotions should be socialized are decidedly different from beliefs about *value or harmfulness* of children’s emotions, so two studies adapted this measure to examine teachers’ value-related beliefs and socialization behaviors with children. With a small sample of prospective teachers, accepting beliefs about children’s emotions were positively associated with their labeling of children’s emotions during teacher-child interactions (Swartz and McElwain, 2012), a teaching activity known to increase emotion understanding skill (Dunsmore and Karn, 2001). In a second study, teachers’ belief that teachers should protect children from negative emotions was associated with their devaluing or ignoring children’s emotion (Ornaghi et al., 2019), behaviors found to suppress emotion understanding in children (Katz et al., 2012). These findings suggest two important types of emotion beliefs, which were also identified in qualitative work of teachers’ beliefs about their *own* expression of negative emotions (Jiang et al., 2019), and a variety of qualitative and quantitative assessments of parental beliefs (Halberstadt et al., 2008, 2013; Parker et al., 2012). Although many beliefs have been studied in the parenting literature, these two beliefs have been found to support a variety of parenting behaviors impacting children’s outcomes (e.g., Denham and Kochanoff, 2002; Halberstadt et al., 2008, 2013; Stelter and Halberstadt, 2011; Lozada et al., 2016). Altogether, these studies converge on the utility of exploring teachers’ beliefs about emotions being valuable or useful and/or emotions being problematic or harmful.

### Teachers’ Emotion Recognition Ability

Despite its importance, attending to and identifying students’ emotions is challenging while also monitoring the myriad of co-occurring classroom events and maintaining classroom momentum toward planned lessons. Yet, we know of no extant work that assesses precursors of teachers’ emotion recognition ability. There is, however, a small body of work relating emotion-related beliefs to recognition skill in the parenting literature. In one study, parents’ belief in the value of negative emotions had little impact, but their belief that negative emotions were problematic or even dangerous was negatively associated with their skill in labeling negative emotions during conversations with their children (Lozada et al., 2016). However, individuals who believed that negative emotions were dangerous seemed to recognize negative facial cues very early in an expression and but then quickly disengaged from those cues, so as to avoid them (Dennis and Halberstadt, 2013).

These studies suggest that beliefs about emotions may be associated with emotion recognition and in a variety of ways. Within the context of the classroom, we thought that teachers who value an emotion (e.g., anger) would be more attentive to the presence of expressions of that emotion, welcoming it as useful information, and, thus, would develop accuracy in assessing emotion-related facial cues. This might be particularly true of emotions about which some ambivalence might be expected, with some teachers preferring to avert their eyes from mildly angry expression and others moving toward investigating further. Thus, in this study, we predicted that teachers who value anger as a useful emotion would be more accurate at recognizing expression of that emotion. Given less clear findings regarding the belief that negative emotions are harmful, we were less sure of a prediction. Teachers who think of anger as problematic or harmful might try to ignore mild expressions of anger, whereas others might be more vigilant about them. Extrapolating from the Ornaghi et al. (2019) finding with teachers and what might be attentional avoidance found in Dennis and Halberstadt (2013), we tentatively predicted that teachers who believe that students’ anger is harmful might ignore mild anger, and, over time, might develop less accuracy at specifying expression of that emotion.

### Current Study

To explore associations between teachers’ beliefs about anger and their emotion recognition of children’s angry facial expressions, teachers completed a questionnaire assessing their beliefs about students’ anger, and participated in judging facial expressions in a dynamic emotion recognition task. We hypothesized that teachers’ beliefs valuing anger would be associated with their skill in recognizing anger expression in children. We tentatively hypothesized that teachers’ beliefs about the harmful nature of anger would be associated with having less skill in recognizing anger expression in children.

## Method

### Participants

Participants were 43 fourth- and fifth-grade teachers from seven public elementary schools within four different county districts in the southeastern United States. Of these, 21 teachers were teaching 4th grade, 21 were teaching 5th grade, and one teacher was responsible for a mixed 4th/5th grade class. Teachers self-identified as White (83.7%), Black (9.3%), Hispanic (4.7%), or East Asian (2.3%); mean age was 37.42 years (SD = 9.49 years) and 86% of the sample was female.

### Procedure

After obtaining district and school principal approval, we invited teachers to participate, and all 43 teachers in the participating schools provided consent. Teachers completed a web-based survey, which included the emotion recognition task and the measure of teachers’ beliefs about anger. Teachers were compensated $80 for the completion of the entire session.

### Measures

#### Perceptions of Children’s Emotions in Videos, Evolving and Dynamic (PerCEIVED) Task (Halberstadt et al., unpublished)

This task assesses emotion recognition of the dynamic facial expressions of 72 children (1/2 Black; 1/2 female). The measure was created so that each expression begins with a neutral face and evolves until an intensity threshold set by FACS coding (Ekman and Friesen, 1978) is met. That is, the first round starts at neutral and then includes slight muscle movement toward the intended expression (“A” intensity); the second round starts at neutral and includes a bit more muscle movement (“B” intensity, and so forth until round 5, which is the apex (height) of the intended emotional expression. The neutrality, prototypically, and intensity of these expressions were validated by two independent, FACS-certified coders (using FACS coding, Ekman et al., 2002). For the current study, we provided just the first and second rounds to the teachers, as these rounds are most analogous to children’s emotional expressions in real life which tend to be fragmented or masked by late elementary school (Camras et al., 2017; Castro et al., 2018).

Each participant was shown one emotional expression at a time, in the form of a video clip, and asked to select which emotion out of six (happy, sad, angry, fear, surprise, and disgust) they thought the child was displaying. Each video was presented only once and in random order, the videos began automatically, and the participants were not able to replay the videos. Thus, the task mimics real-life experience in that the facial expressions of children in the age group served by the participating teachers tend to be fleeting and occur in somewhat fragmented forms (Castro et al., 2018). Accurate selections were labelled recoded as “1” and inaccurate selections as “0.” For the current study, only accuracy for anger was analyzed, resulting in 24 videos (3 children in each race/gender/emotion group/round).

Stability in skill level across the first three rounds in this task over a 3-month period is strong (*r* = 0.70, *p* < 0.001) as is convergent validity with two other emotion recognition tasks (*r*s = 0.47 and 0.49, *p*s < 0.001); Halberstadt et al., unpublished). Although this task is new, there is some construct validity in that the task with three rounds reveals racialized anger bias in a sample of preservice teachers (Halberstadt et al., unpublished), as predicted^1^.

#### Teachers’ Everyday Beliefs About Student Anger (TBASE - Anger) Questionnaire (Hagan et al., unpublished)

To assess teachers’ beliefs, we created 30 items based on the parental belief literature (particularly Halberstadt et al., 2013; see Appendix A), as originally inspired by Hyson and Lee (1996), but focused on the value of emotion and teachers’ emotional responsiveness to others’ emotions. An exploratory factor analysis with a separate sample of teachers (*N* = 225) produced four factors (Hagan et al., unpublished). To capture the two beliefs of interest for this study, we used the scales “Anger is Useful” (seven items, e.g., “It is useful for children to feel angry sometimes” and “Anger can help me understand what the student is thinking”; α = 0.77) and “Anger is Harmful” (six items; e.g., “Children can think more clearly when anger does not get in the way” and “Anger in children can be emotionally dangerous”; α = 0.72). Teachers responded to the items using a Likert-type scale (1 = “not at all true” to 5 = “very often true”); scores were averaged within scale.

#### Covariates

We included teacher age as a proxy for teacher experience with children. Although not a pure proxy for teaching experience, the current sample did not include a measure of years spent in the classroom. We also controlled for teacher gender because emotion-related beliefs sometimes vary by gender in the parenting literature. Although our sample included only six male teachers, each teacher reported 24 unique pieces of information (2 rounds containing 12 children each), and MLM nested designs can support this level of gender imbalance. Finally, “round” was entered as a covariate to statistically control for the linear effects of learning between round one and round two, as the emotional expression intensifies.

## Results

### Analytical Plan

We began by examining descriptive statistics and correlations for the TBASE and anger recognition. Then, to address whether teachers’ beliefs were associated with their accuracy, we ran two (anger is useful, anger is harmful) multilevel logistic models. Based on the correlations in the descriptive analyses, we entered teacher age, teacher gender, and task round (to control for learning effects) as covariates. To test our multilevel logistic models we used SAS software, Version 9.4. All assumptions regarding univariate and multivariate normality were met. All results are described in terms of odds ratios; “1” indicates and accuracy emotional label for anger and “0” indicates an inaccurate response.

Preliminary multilevel analyses began with a fully unconditional model of accuracy for anger and included only the intercept (accuracy) to partition the variance between within and between person effects. The ICC calculation indicated that 7% of the variance was attributable to between-person differences and 93% of the variance was attributable to within-person variance. The null model indicated that the average odds of accurately identifying anger in children was 0.63 (CI = 0.65, 0.84) and was significant.

Level 1: ACCURACY_it_ = β_0it_ + β_1_(BELIEF)_it_ + β_2_ (TEACHER AGE)_it_ + β_3_(TEACHER GENDER)_it_+ β_4_(ROUND)_it_ + r_it_
Level 2: β_0i_ = γ_00_
  β_1i_ = *γ*_*10*_
  β_2i_ = *γ*_*20*_
  β_3i_ = *γ*_*30*_
  β_4i_ = *γ*_*40*_

In these equations, the within-person effects at Level 1 are modeled by the main effect of Teacher’s beliefs about anger (β_1_), and the covariates: teacher age (β_2_), teacher gender (β_3_), and the linear effects of round (to statistically control for increases in accuracy as the expression of the emotion intensifies) (β_4_).

### Descriptive Analyses

As shown in Table 1, the teachers reported beliefs that averaged slightly above the midpoint for both scales and showed variability across individuals. The two beliefs were independent (*r* = 0.08), consistent with previous work with parents (e.g., Halberstadt et al., 2008), and despite the use of factor analyses that allowed for some degree of overlap between the scales (Hagan et al., unpublished). The average individual accuracy for facial anger was 0.43, suggesting skill well above chance (0.167), but also not representing a high level of accuracy. Neither the belief that anger was harmful (*r* = −0.13), nor the belief that anger was useful (*r* = 0.01) was correlated with teachers’ age. Gender mattered in that male teachers were more likely to endorse the belief that anger was useful (*M* = 3.78, *SD* = 0.15) than female teachers (*N* = 37; *M* = 3.35, *SD* = 0.38), *t*(41) = 2.73, *p* < 0.05; there were no gender differences, however, for the belief that anger was harmful.

**TABLE 1:** Descriptive statistics for teacher beliefs.

	Descriptives		
	M	SD	Range
Belief: Anger is Useful	3.41	0.39	2.57–4.29
Belief: Anger is Harmful	3.42	0.40	2.33–4.17

*The possible range for the two beliefs was 1 to 5.*

## Do Teachers’ Beliefs About Anger Predict Anger Accuracy?

Table 2 reports the odds ratios and confidence intervals for teacher’s beliefs about anger as predictors of anger accuracy. Consistent with the parenting literature, teachers’ belief that anger is useful was a significant predictor of accuracy in identifying anger expressions, even when accounting for the covariates of teacher age, teacher gender, and round. Teachers’ belief that anger is harmful was not associated with anger accuracy.

**TABLE 2:** Teachers’ beliefs about emotion predicting anger expression recognition.

	Outcome: Anger Expression Recognition			
	Anger is Useful		Anger is Harmful	
	OR	CI	OR	CI
Intercept	0.10	0.02, 0.78	1.00	0.14, 7.40
Teacher Belief	1.82*	1.08, 3.05	0.96	0.59, 1.55
Covariate				
Teacher Age	0.98*	0.96, 0.99	0.98	0.96, 1.00
Teacher Gender	1.12	0.63, 1.98	0.86	0.49, 1.50
Round	1.41**	1.09, 1.83	1.41**	1.09, 1.83

Gender: “0” = Male, “1” = Female. For odds ratios, values above 1 indicate probability of anger accuracy occurring, and values below 1 indicate probability of anger accuracy not occurring. *p < 0.05, **p < 0.01.

Teacher age was a significant covariate in the belief that anger is useful but not harmful model, reaching significance at *p* = 0.05. We found this interesting because teacher age was not initially a significant correlate of anger beliefs. Therefore, we ran one additional, *post hoc* model with teacher age predicting anger accuracy (controlling for round) and discovered age was not a significant predictor of anger expression accuracy (*p* = 0.07).

## Discussion

Previous research demonstrates that everyday beliefs about emotion play a role in orienting and motivating individuals’ attention to and skill in understanding others’ emotions (Dennis and Halberstadt, 2013; Castro et al., 2015; Ford and Gross, 2018). However, few studies, if any, explore how beliefs about specific emotions relate to recognition of emotion expressions related to those emotions, especially among teachers. As hypothesized, the belief that anger is useful was associated with teachers’ skill in recognizing children’s anger expressions as they were forming.

In contrast, teachers’ belief that anger is harmful was not associated with skill in identifying anger expressions. It may be that the belief that anger is harmful has no impact on recognition of anger expressions. However, it may also be that some teachers who think of anger as problematic or harmful might avoid thinking about anger, whereas others might be more vigilant about identifying anger early in its development, and these two orientations might cancel out the effects entirely. Because both of these strategies might have merit in the classroom (letting mild anger be expressed and dissipated without responding to it or maintaining early vigilance and resolution), we think continued research about this belief is warranted. Eye-tracking may be particularly useful for gauging how teachers who hold different beliefs (e.g., emotions are of value or are harmful) search faces to identify emotions and engage with or avoid information that is readily available. If patterns do emerge in laboratory studies, next steps might include how beliefs then impact teachers’ responses to anger in the classroom.

Overall, our findings suggest that at least some emotion-related beliefs are involved in processes that lead to actual skill and open up a range of possibilities. Future research can explore the pathways and mechanisms of everyday beliefs that create skill in recognizing emotion expression. Although we found that belief in the usefulness of anger is associated with skill in recognizing expressions associated with anger, we still do not know if belief in the value of emotions *in general* is sufficient for all emotion recognition, or if emotion recognition is principally reliant on beliefs specifically valuing discrete emotions. It may also be that the beliefs that teachers and others hold about often-maligned emotions such as anger may be more important for emotion recognition than beliefs about more generally appreciated emotions such as joy and compassion. Given the overall lack of coherence between experiencing and expressing anger in children and adults (Reisenzein et al., 2013; Castro et al., 2018), these teachers may have a “head start” on recognizing what is experienced in the minds of children. Given that lack of coherence, however, skilled teachers may want to invite conversation and confirmation about what children are actually feeling. Another possible avenue for research is to explore how anger beliefs affect other skills comprising emotion understanding, such as emotion knowledge, which involves awareness of the relevant causes for anger, the trajectory of emotion experience in terms of build-up and dissipation, and consequences to the individual and the group when anger is expressed (Castro et al., 2016). Because we now know that at least one anger belief relates to anger recognition, we wonder whether this anger belief and others might relate to increased knowledge of or skill guiding others toward emotion regulation. Certainly, the motivational role that everyday beliefs play suggests that beliefs about emotion could be a motivational force to building skills that encompass emotion understanding.

Our exploratory study certainly has its limitations. Although multi-level modeling is robust with small samples, the belief that anger is harmful might have achieved significance with a larger sample and more statistical power. Further, with only 12 different actors displaying anger and with FACS-coded rules for prototypicality, it would surely be advisable to replicate by incorporating displays of felt anger in the classroom and at different ages, thus increasing representation of a variety of expressions of anger, and investigating how different beliefs might matter when working with students from different developmental periods. We also acknowledge that teachers in our study were tasked with identifying only facially expressed anger. Clearly, vocal, bodily, behavioral, situational, and physiological cues provide important and distinct emotional information that allow for holistic judgments about emotions (Yeh et al., 2016), especially for anger (Cacioppo et al., 2000). In fact, these different modalities of emotion expression may be particularly important cues for some emotions (Schirmer and Adolphs, 2017).

Although our study included teachers from seven different elementary schools and four different districts, and thus, has some generalizability, we lacked the sample size to examine whether school emphasis on socio-emotional learning could influence teachers’ beliefs about anger or anger recognition. Because socio-emotional training is very under-represented in teacher education programs and continued education (Schonert-Reichl et al., 2017; Garner et al., 2018), the differential attention to socio-emotional skills that is then fostered within schools and school districts may create systemic, macro-level differences in teachers’ own emotion-related belief systems and skills as well as those of their students (Bronfenbrenner, 1979; Garbarino et al., 2005; von der Embse et al., 2016).

We note that teachers may also differ with regard to whether they view students’ emotions as destructive or constructive. Broadly speaking, these variations have to do with the extent to with students’ emotions and emotion-related behaviors are perceived as being beneficial to teachers’ classroom and instructional goals (Frenzel, 2014). Because we did not contextualize students’ emotions in our belief measure, our findings may underestimate associations between teachers’ emotion beliefs and their anger recognition. That is, teachers’ ability and willingness to respond to students’ anger with a fair, calm, reasoned approach may be dependent upon their causal attributions of student anger, an element that we did not consider.

Finally, although many studies demonstrate the utility of emotion recognition skill in interpersonal relationships, business, and medical settings (DiMatteo et al., 1986; Byron et al., 2007; Gollan et al., 2010; Mier et al., 2010; Hall, 2011; Israelashvili et al., 2020), we do not actually know whether teachers’ emotion recognition also provides benefit to them and their students, thus this is a limitation within our study. An important next step would be to test whether teachers’ beliefs about anger impact teachers’ skill in recognizing children’s anger expressions and experiences in the classroom and teachers’ effective responses to their students. For example, it would be useful to know whether teachers who have value-oriented beliefs about anger, and/or who can recognize early-forming anger expressions (as in Rounds 1 and 2 with only partial expressions), are better able to facilitate students’ emotion regulation by identifying and guiding students’ expression and experience more effectively. It would also be ideal if teachers could use early recognition of facial expressions to better scaffold lessons that do not overwhelm students with emotions detrimental to learning. Whether they do or not are important testable questions.

Despite these limitations, our findings suggest the importance of studying specific beliefs about the value of emotion, and in the understudied context of teaching. In so doing, this study underscores the potential of research studying emotion-related beliefs in the development of emotion-related skills of educators, and most likely adults in general. We hope that our study, with both a new conceptualization about teachers’ beliefs about the value and harm associated with anger and a measurement tool with which to assess those two beliefs, will invite further exploration of beliefs about anger and their outcomes at both the individual and organizational levels in educational settings.

## Data Availability Statement

The datasets generated for this study are available on request to the corresponding author.

## Ethics Statement

The studies involving human participants were reviewed and approved by North Carolina State University IRB Board. Written informed consent for participation was not required for this study in accordance with the national legislation and the institutional requirements.

## Author Contributions

CH and AH conceived of the present study idea. AH obtained district, school, and teacher consents and coordinated data collection. CH and AC also provided considerable support for data collection, including developing the qualtrics programs that teachers used. AC helped CH with the analyses to answer the research questions and provided edits to the results section. PG contributed substantially to the introduction and provided edits and critical feedback throughout the manuscript. CH took the lead in writing the manuscript with substantial editing from AH.

## Conflict of Interest

The authors declare that the research was conducted in the absence of any commercial or financial relationships that could be construed as a potential conflict of interest.

## Footnotes

1 The PerCEIVED task also allows for the assessment of anger bias, which is the tendency to perceive anger when it does not exist (e.g., when the participant selects anger when the child is displaying FACS-coded disgust). In analyses, we did not find either belief associated with anger bias (both *p*s > 0.05), suggesting that teachers’ beliefs about anger did not lead to a propensity toward misperceiving anger in a general way (see Supplementary Table S1).

## References

[B1] Averill J. (1982). *Anger and Aggression: An Essay on Emotion.* New York, NY: Springer-Verlag.

[B2] Barrett L. F. Adolphs R. Marsella S. Martinez A. M. Pollak S. D. (2019). Emotional expressions reconsidered: challenges to inferring emotion from human facial movements. *Psychol. Sci. Public Interest* 20 1–68. 10.1177/1529100619832930 31313636 PMC6640856

[B3] Bronfenbrenner U. (1979). *The Ecology of Human Development.* Cambridge, MA: Harvard University Press.

[B4] Byron K. Terranova S. Nowicki S. Jr. (2007). Nonverbal emotion recognition and salespersons: linking ability to perceived and actual success. *J. Appl. Soc. Psychol.* 37 2600–2619. 10.1111/j.1559-1816.2007.00272.x

[B5] Cacioppo J. T. Berntson G. G. Larsen J. T. Poehlmann K. M. Ito T. A. (2000). “The psychophysiology of emotion,” in *Handbook of Emotions*, eds Lewis R. Haviland-Jones J. M. (New York, NY: Guilford), 173–191.

[B6] Camras L. A. Castro V. L. Halberstadt A. G. Shuster M. M. (2017). “Spontaneously produced facial expressions in infants and children,” in *The Science of Facial Expression*, eds Fernández-Dols J. M. Russell J. A. (New York, NY: Oxford University Press).

[B7] Castro V. L. Camras L. A. Halberstadt A. G. Shuster M. (2018). Children’s prototypic facial expressions during emotion-eliciting conversations with their mothers. *Emotion* 18 260–276. 10.1037/emo0000354 28714700 PMC5771990

[B8] Castro V. L. Cheng Y. Halberstadt A. G. Grühn D. (2016). EUReKA! A conceptual model of emotion understanding. *Emot. Rev.* 8 258–268. 10.1177/1754073915580601 27594904 PMC5006746

[B9] Castro V. L. Halberstadt A. G. Lozada F. T. Craig A. B. (2015). Parents’ emotion-related beliefs, behaviors, and skills predict children’s recognition of emotion. *Infant Child Dev.* 24 1–22. 10.1002/icd.1868 26005393 PMC4437215

[B10] De Castella K. Platow M. J. Tamir M. Gross J. J. (2018). Beliefs about emotion: implications for avoidance-based emotion regulation and psychological health. *Cogn. Emot.* 32 773–795. 10.1080/02699931.2017.1353485 28737108

[B11] Demetriou H. Wilson E. (2010). Children should be seen and heard: the power of student voice in sustaining new teachers. *Improv. Sch.* 13 54–69. 10.1177/1365480209352545

[B12] Denham S. Kochanoff A. T. (2002). Parental contributions to preschoolers’ understanding of emotion. *Marriage Fam. Rev.* 34 311–343. 10.1300/J002v34n03_06

[B13] Dennis P. A. Halberstadt A. G. (2013). Is believing seeing? The role of emotion-related beliefs in selective attention to affective cues. *Cogn. Emot.* 27 3–20. 10.1080/02699931.2012.680578 22712535

[B14] DiMatteo M. R. Hays R. D. Prince L. M. (1986). Relationship of physicians’ nonverbal communication skill to patient satisfaction, appointment noncompliance, and physician workload. *Health Psychol.* 5 581–594. 10.1037/0278-6133.5.6.581 3803351

[B15] Dunsmore J. C. Her P. Halberstadt A. G. Perez-Rivera M. B. (2009). Parents’ beliefs about emotions and children’s recognition of parents’ emotions. *J. Nonverbal Behav.* 33 121–140. 10.1007/s10919-008-0066-6 20160992 PMC2755301

[B16] Dunsmore J. C. Karn M. A. (2001). Mothers’ beliefs about feelings and children’s emotional understanding. *Early Educ. Dev.* 12 117–138. 10.1207/s15566935eed1201_7

[B17] Ekman P. Friesen W. V. (1978). *Facial Action Coding System: Investigator’s Guide Part I.* Palo Alto, CA: Consulting Psychologist Press.

[B18] Ekman P. Friesen W. V. Hager J. (2002). *The Facial Action Coding System*, 2nd Edn. Salt Lake City, UT: Research Nexus.

[B19] Ford B. Q. Gross J. J. (2018). Emotion regulation: why beliefs matter. *Can. Psychol.* 59 1–14. 10.1037/cap0000142

[B20] Frenzel A. C. (2014). “Teacher emotions,”,” in *Educational Psychology Handbook series International Handbook of Emotions in Education*, eds Pekrun R. Linnenbrink-Garcia L. (New York, NY: Routledge/Taylor Francis Group), 494–518.

[B21] Garbarino J. Bradshaw C. P. Kostelny K. (2005). “Neighborhood and community influences on parenting,” in *Parenting: An Ecological Perspective*, eds Luster T. Okagaki L. (Mahwah: Erlbaum), 297–318.

[B22] Garner P. W. Bender S. L. Fedor M. (2018). Mindfulness−based SEL programming to increase preservice teachers’ mindfulness and emotional competence. *Psychol. Sch.* 55 377–390. 10.1002/pits.22114

[B23] Garner P. W. Bolt E. Roth A. N. (2019a). Emotion-focused curricula models and expressions of and talk about emotions between teachers and young children. *J. Res. Child. Educ.* 33 180–193. 10.1080/02568543.2019.1577772

[B24] Garner P. W. Parker T. S. Prigmore S. B. (2019b). Caregivers’ emotional competence and behavioral responsiveness as correlates of early childcare workers’ relationships with children in their care. *Infant Ment. Health J.* 40 496–512. 10.1002/imhj.21784 31090951

[B25] Gollan J. K. McCloskey M. Hoxha D. Coccaro E. F. (2010). How do depressed and healthy adults interpret nuanced facial expressions? *J. Abnorm. Psychol.* 119 804–810. 10.1037/a0020234 20939654 PMC3805828

[B26] Halberstadt A. G. Dunsmore J. C. Bryant A. Jr. Parker A. E. Beale K. S. Thompson J. A. (2013). Development and validation of the parents’ beliefs about children’s emotions questionnaire. *Psychol. Assess.* 25 1195–1210. 10.1037/a0033695 23914957 PMC4018216

[B27] Halberstadt A. G. Thompson J. A. Parker A. E. Dunsmore J. C. (2008). Parents’ emotion−related beliefs and behaviours in relation to children’s coping with the 11 September 2001 terrorist attacks. *Infant Child Dev.* 17 557–580. 10.1002/icd.569

[B28] Hall J. A. (2011). Clinicians’ accuracy in perceiving patients: its relevance for clinical practice and a narrative review of methods and correlates. *Patient Educ. Couns.* 84 319–324. 10.1016/j.pec.2011.03.006 21592718

[B29] Hall J. A. Andrzejewski S. A. Yopchick J. E. (2009). Psychosocial correlates of interpersonal sensitivity: a meta-analysis. *J. Nonverbal Behav.* 33 149–180. 10.1007/s10919-009-0070-5

[B30] Hargreaves A. (2000). Mixed emotions: teachers’ perceptions of their interactions with students. *Teach. Teach. Educ.* 16 811–826. 10.1016/S0742-051X(00)00028-7 25141091

[B31] Hyson M. C. Lee K.-M. (1996). Assessing early childhood teachers’ beliefs about emotions: content, contexts, and implications for practice. *Early Educ. Dev.* 7 59–78. 10.1207/s15566935eed0701_5

[B32] Israelashvili J. Sauter D. A. Fischer A. H. (2020). Different faces of empathy: feelings of similarity disrupt recognition of negative emotions. *J. Exp. Soc. Psychol.* 87 103912. 10.1016/j.jesp.2019.103912 32127724 PMC7001982

[B33] Jiang J. Vauras M. Volet S. Salo A. E. (2019). Teacher beliefs and emotion expression in light of support for student psychological needs: a qualitative study. *Educ. Sci.* 9 68 10.3390/educsci9020068

[B34] Katz L. F. Maliken A. C. Stettler N. M. (2012). Parental meta−emotion philosophy: a review of research and theoretical framework. *Child Dev. Perspect.* 6 417–422. 10.1111/j.1750-8606.2012.00244.x

[B35] Leonard D. J. Moons W. G. Mackie D. M. Smith E. R. (2011). “We’re mad as hell and we’re not going to take it anymore”: anger self-stereotyping and collective action. *Group Process. Intergroup Relat.* 14 99–111. 10.1177/1368430210373779

[B36] Lozada F. T. Halberstadt A. G. Craig A. B. Dennis P. A. Dunsmore J. C. (2016). Parents’ beliefs about children’s emotions and parents’ emotion-related conversations with their children. *J. Child Fam. Stud.* 25 1525–1538. 10.1007/s10826-015-0325-1

[B37] Mayer J. D. Salovey P. Caruso D. R. Sitarenios G. (2001). Emotional intelligence as a standard intelligence. *Emotion* 1 232–242. 10.1037/1528-3542.1.3.232 12934682

[B38] Mier D. Lis S. Neuthe K. Sauer C. Esslinger C. Gallhofer B. (2010). The involvement of emotion recognition in affective theory of mind. *Psychophysiology* 47 1028–1039. 10.1111/j.1469-8986.2010.01031.x 20456660

[B39] Miller P. Sperry L. (1987). The socialization of anger and aggression. *Merrill Palmer Q.* 33 1–31.

[B40] Oertwig D. Riquelme E. Halberstadt A. G. (2019). Respect and fear: socialization of children’s fear among the mapuche people of Chile. *Cult. Brain* 7 212–238. 10.1007/s40167-019-00077-y

[B41] Ornaghi V. Agliati A. Pepe A. Gabola P. (2019). Patterns of association between early childhood teachers’ emotion socialization styles, emotion beliefs and mind-mindedness. *Early Educ. Dev.* 31 47–65. 10.1080/10409289.2019.1627805

[B42] Parker A. E. Halberstadt A. G. Dunsmore J. C. Townley G. E. Bryant A. Jr. Thompson J. A. (2012). “Emotions are a window into one’s heart”: a qualitative analysis of parental beliefs about children’s emotions across three ethnic groups. *Monogr. Soc. Res. Child Dev.* 77 1–144. 22905794 10.1111/j.1540-5834.2012.00676.x

[B43] Reisenzein R. Studtmann M. Horstmann G. (2013). Coherence between emotion and facial expression: evidence from laboratory experiments. *Emot. Rev.* 5 16–23. 10.1177/1754073912457228

[B44] Schirmer A. Adolphs R. (2017). Emotion perception from face, voice, and touch: comparisons and convergence. *Trends Cogn. Sci.* 21 216–228. 10.1016/j.tics.2017.01.001 28173998 PMC5334135

[B45] Schonert-Reichl K. A. Kitil M. J. Hanson-Peterson J. (2017). *To Reach the Students, Teach the Teachers: A National Scan of Teacher Preparation and Social Emotional Learning. a Report Prepared for the Collaborative for Academic, Social, and Emotional Learning.* Chicago, IL: CASEL.

[B46] Stelter R. L. Halberstadt A. G. (2011). The interplay between parental beliefs about children’s emotions and parental stress impacts children’s attachment security. *Infant Child Dev.* 20 272–287. 10.1002/icd.693 21731472 PMC3124851

[B47] Swartz R. A. McElwain N. L. (2012). Preservice teachers’ emotion-related regulation and cognition: associations with teachers’ responses to children’s emotions in early childhood classrooms. *Early Educ. Dev.* 23 202–226. 10.1080/10409289.2012.619392

[B48] Tavris C. (1982). *Anger: The Misunderstood Emotion.* New York, NY: Simon Schuster.

[B49] Valente S. Monteiro A. P. Lourenço A. A. (2019). The relationship between teachers’ emotional intelligence and classroom discipline management. *Psychol. Sch.* 56 741–750. 10.1002/pits.22218

[B50] von der Embse N. P. Pendergast L. L. Segool N. Saeki E. Ryan S. (2016). The influence of test-based accountability policies on school climate and teacher stress across four states. *Teach. Teach. Educ.* 59 492–502. 10.1016/j.tate.2016.07.013

[B51] Yeh P. W. Geangu E. Reid V. (2016). Coherent emotional perception from body expressions and the voice. *Neuropsychologia* 91 99–108. 10.1016/j.neuropsychologia.2016.07.038 27480921

[B52] Yelinek J. Grady J. S. (2019). ‘Show me your mad faces!’Preschool teachers’ emotion talk in the classroom. *Early Child Dev. Care* 189 1063–1071. 10.1080/03004430.2017.1363740

