# Supplementary material for: Teachers’ Beliefs About Children’s Anger and Skill in Recognizing Children’s Anger Expressions
Source: Front Psychol. 2020 Mar 24;11:474. doi: 10.3389/fpsyg.2020.00474 (PMC7107660; doi:10.3389/fpsyg.2020.00474)
Supplement: Supplementary file 1 [file Image_1.pdf]

Appendix A

Teachers' Beliefs about Student Emotions – Anger

(TBASE – Anger)

Directions: For this questionnaire, please indicate how much you agree with each statement or to what degree you believe each statement to be true.

1	2	3	4	5
Not at all true	Rarely true	Sometimes true	Often true	Very often true

<u>Harmful</u>

When students get angry, they will have a hard time learning.
Children can think more clearly when anger does not get in the way.
A student's anger can harm their peer relationships.
It is important in school for children to avoid feeling angry whenever possible.
Anger in children can be emotionally dangerous.
A student's anger often leads to negative outcomes in the classroom.

<u>Useful</u>

It is useful for children to feel angry sometimes.
A student's anger can be a relief to them, like a storm that clears the air.
Anger can help students find a way to change a situation that needs changing.
It is good for children to let their anger out.
Expressing anger is a good way for a student to let their desires and opinions be known.
Being angry can motivate students to change or fix something in their lives.
Anger can help me understand what the student is thinking.

<u>Manipulate</u>

Children use emotions to manipulate others.
Children often act sad or angry just to get their own way.
When students are angry at a teacher, they are usually challenging the teacher's decisions.

<u>Distressing</u>

When a child gets angry in the classroom, I get frustrated with them.
I am uncomfortable when a student shows anger in my classroom.
When children show anger, they usually make the situation worse
I try to view a child's anger as an opportunity instead of a problem. (REVERSAL)
