# Supplementary material for: Teachers’ Beliefs About Children’s Anger and Skill in Recognizing Children’s Anger Expressions
Source: Front Psychol. 2020 Mar 24;11:474. doi: 10.3389/fpsyg.2020.00474 (PMC7107660; doi:10.3389/fpsyg.2020.00474)
Supplement: Supplementary file 2 [file Table_1.pdf]

Table S1. Teachers' beliefs about emotion predicting anger bias

	Outcome: Anger Bias			
	Anger is Useful		Anger is Harmful	
	OR	CI	OR	CI
Intercept	0.37	0.06, 2.17	0.16*	0.04, 0.72
Teacher Belief	1.09	0.71, 1.68	1.39	0.96, 2.01
Covariate				
Teacher Age	0.98	0.97, 1.00	0.99	0.97, 1.00
Teacher Gender	0.85	0.53, 1.35	0.82	0.54, 1.23
Round	0.53**	0.43, 0.64	0.53**	0.43, 0.64

Note:
1. Gender: "0" = Male, "1" = Female.
2. For odds ratios, values above 1 indicate probability of anger bias occurring, and values below 1 indicate probability of anger bias not occurring.
*$p < .05$, **$p < .01$
